# Age effects on basic symptoms in the community: A route to gain new insight into the neurodevelopment of psychosis?

**DOI:** 10.1007/s00406-018-0949-4

**Published:** 2018-10-25

**Authors:** Frauke Schultze-Lutter, Stephan Ruhrmann, Chantal Michel, Jochen Kindler, Benno G. Schimmelmann, Stefanie J. Schmidt

**Affiliations:** 1 University Hospital of Child and Adolescent Psychiatry and Psychotherapy, University of Bern, Bern, Switzerland; 2 Department of Psychiatry and Psychotherapy, Medical Faculty, Heinrich-Heine-University, Bergische Landstrasse 2, 40629 Düsseldorf, Germany; 3 Department of Psychiatry and Psychotherapy, University of Cologne, Cologne, Germany; 4 University Hospital of Child and Adolescent Psychiatry, University Hospital Hamburg-Eppendorf, Hamburg, Germany; 5 Department of Clinical Psychology and Psychotherapy, University of Bern, Bern, Switzerland

**Keywords:** Psychosis, Brain development, Neuropsychopathology, Basic symptoms, Age, Epidemiology

## Abstract

Reports of limited clinical significance of attenuated psychotic symptoms before age 15/16 indicate an important role of neurodevelopment in the early detection of psychoses. Therefore, we examined if age also exerts an influence on the prevalence and clinical significance of the 14 cognitive and perceptive basic symptoms (BS) used in psychosis-risk criteria and conceptualized as the most direct self-experienced expression of neurobiological aberrations. A random representative general population sample of the Swiss canton Bern (*N* = 689, age 8–40 years, 06/2011–05/2014) was interviewed for BS, psychosocial functioning, and current mental disorder. BS were reported by 18% of participants, mainly cognitive BS (15%). In regression analyses, age affected perceptive and cognitive BS differently, indicating an age threshold for perceptive BS in late adolescence (around age 18) and for cognitive BS in young adulthood (early twenties)—with higher prevalence, but a lesser association with functional deficits and the presence of mental disorder in the below-threshold groups. Thereby, interaction effects between age and BS on functioning and mental disorder were commonly stronger than individual effects of age and BS. Indicating support of the proposed “substrate-closeness” of BS, differential age effects of perceptual and cognitive BS seem to follow normal brain maturation processes, in which they might occur as infrequent and temporary non-pathological disturbances. Their persistence or occurrence after conclusion of main brain maturation processes, however, might signify aberrant maturation or neurodegenerative processes. Thus, BS might provide important insight into the pathogenesis of psychosis and into differential neuroprotective or anti-inflammatory targets.

**Electronic supplementary material:**

The online version of this article (10.1007/s00406-018-0949-4) contains supplementary material, which is available to authorized users.

## Introduction

Psychoses, in particular the schizophrenia spectrum, are considered a fundamentally neurodevelopmental disorder involving two critical time windows—early (perinatal) brain development and adolescence—that together produce the symptomatic manifestations of the disorder [1]. Thus, following the increasing involvement of younger age groups in the early detection of psychotic disorders [2–5], concerns have been voiced about the likely impact of age, i.e., developmental aspects, on the prevalence, clinical significance, and psychosis-predictive value of risk criteria and symptoms [6]. These have been corroborated by a recent meta-analysis of conversion-to-psychosis rates that reported significantly lower rates in ultra-high-risk (UHR) samples comprising exclusively children and adolescents compared to adult or mixed adult-adolescent samples [5].

## Studies of age effects on attenuated psychotic symptoms

The attenuated psychotic symptom (APS) criterion is the main UHR criterion [4, 5]. For the APS criterion as well as for APS themselves, an effect of age on the prevalence and clinical significance was recently reported from clinical and community studies [7–10] with the exception of one study of patients with 22q11 deletion syndrome [11]. These studies, including one on the same sample as the present one [8], indicate that APS, in particular perception-related APS, are more prevalent in samples below age 15/16, while at the same time they have less clinical significance in terms of a weaker association with functional impairments and/or mental disorder, incl. subsequent development of psychosis, below this age threshold [7–10]. The only negative study on 22q11 deletion syndrome patients [11] was explained by the high genetic liability to develop schizotypal personality features and psychosis, respectively, and, consequently, the lesser involvement of other age-related factors, e.g., development of cognitive abilities, on the occurrence of APS [11].

## The concept of basic symptoms and age-related considerations

With regard to the basic symptom (BS) risk approach [12, 13], age effects have so far been examined and described only with regard to their dimensional structure [14, 15]. BS are subtle, subjectively experienced subclinical disturbances in drive, affect, thinking, speech, (body) perception, motor action, central-vegetative functions, and stress tolerance and can usually be assessed from age 8 onwards [15]. They were conceptualized as the earliest subjectively experienced symptoms of psychosis and the most immediate symptomatic expression of the neurobiological correlates of the illness [12, 13, 16, 17]. Preliminary evidence for neurobiological mechanisms underlying BS indicates that diverse anatomical, pharmacological and functional correlates may be involved in the manifestation of BS in psychotic and risk individuals [16]. For this proposed characteristic of BS as being “substrate-close” [12, p. 646], it must be assumed that BS are influenced by neurodevelopment to an even greater degree than are APS.

By definition, BS differ from what patients consider to be their “normal” mental self, and thus, are distinct from trait-like schizotypy features considered as part of the normal self. Furthermore, BS remain predominately private and are rarely perceivable by others, although patients’ self-initiated coping strategies (including avoidance strategies and social withdrawal) in response to their BS may be observable, e.g., as negative symptoms [13]. Due to their spontaneous, immediate recognition by patients as disturbances of their own (mental) processes, BS are also distinct from APS or frank psychotic symptoms, in which reality testing is already disturbed. Within the BS concept, (attenuated) psychotic symptoms are considered to arise from BS when everyday situations and demands overstrain patients’ already pathologically vulnerable information processing capacity [13]. Thus, given salutary environmental and personal conditions (e.g., a supportive social network; good social, problem solving, and coping skills; or high self-efficacy), BS can be counterbalanced as long as their number and/or severity do not overextend protective factors and patients’ resilience [13].

While most BS may occur in other disorders, especially non-psychotic affective disorders [14, 15], a subset of 14 cognitive and perceptive BS appear to be specific to psychosis. These are included in the two psychosis-risk criteria [2, 5, 14, 18]: cognitive disturbances (COGDIS) and cognitive-perceptive BS (COPER) (Online Resource 1).

## Aims and hypotheses

Following the main method employed to study age effects in APS [8, 9, 11], i.e., comparing predefined age groups, and interactions of symptoms and age by regression analyses, the current study examined potential age effects in the prevalence and clinical significance of the 14 BS included in COPER and COGDIS in the general population. With respect to the age threshold around the age of 15/16 described for APS, we expected an earlier or similar age threshold for BS, with a higher prevalence of perceptive BS and a lesser clinical significance in terms of a weaker association with impaired functioning and presence of mental disorders of cognitive BS in children and young adolescents compared to older adolescents and adults.

## Materials and methods

### Sample

The sample consisted of community participants in two studies approved by the ethics committee of the University of Bern: the Binational Evaluation of At-Risk Symptoms in Children and Adolescents (BEARS-Kid) study [8] and the Bern Epidemiology At-Risk (BEAR) study [8, 19]. In both studies, first contact was established by an information letter on respective study aims, voluntariness of participation, and anonymous use of data in group statistics. For the BEAR study, participation in the telephone interview was then equated with provision of informed consent; for the BEARS-Kid study, written informed assent/consent was secured from participants and their parents.

Stratified sampling by gender (1:1) was used to randomly select potential participants aged 8–17 years in the BEARS-Kid study and 16–40 years in the BEAR study from 384,000 persons in these age groups included in the obligatory population register of Canton Bern, Switzerland. In both studies, eligibility criteria included appropriate age, main residence in Canton Bern, and an available telephone number. Interviews were discontinued if participants had a lifetime diagnosis of psychosis or insufficient German, French, or English language skills. Recruitment and telephone assessments for the BEAR study were conducted over 14 months; recruitment and face-to-face assessments for the BEARS-Kid study over 33 months. Prior to merging data, a feasibility study examining the correspondence of telephone and face-to-face assessments of BS reported excellent concordance rates (78–100%) between these two assessment modes [20], thus indicating that data of both studies could be merged and compared without danger of introducing a systematic assessment bias.

The participation rate of those eligible was 41.5% in the BEARS-Kid study and 66.4% in the BEAR study. In both studies, participants and non-participants did not differ in age, gender, or nationality. Main reasons for refusal were lack of interest in the topic (BEARS-Kid: 49.6%; BEAR: 52.9%) or lack of time (BEARS-Kid: 33.8%; BEAR: 44.5%).

Where allowed by the subsample size, each child/adolescent (aged 8–17 years) was randomly matched by gender and highest parental educational level to one participant of each of the four adult age groups (18–19, 20–24, 25–29, and 30–40 years). Our final sample (*N* = 689) comprised 535 adults and 154 children/adolescents.

### Measures

The Schizophrenia Proneness Instrument, Adult (SPI-A; [21]; in BEAR study) and Child and Youth versions (SPI-CY; [22]; in BEARS-Kid study) were used to assess BS and additional risk criteria requirements for novelty and frequency (Online Resource 1). The definitions of corresponding BS as well as of BS criteria are equal in both versions of the SPI. More specifically, both criteria require that the respective BS occurred at least once per week within the past 3 months (frequency requirement) and had a distinct first occurrence (novelty requirement). As in APS [8–10], for the current analysis, perceptive and cognitive BS were distinguished rather than employ the partially overlapping criteria for BS (Online Resource 1). Perceptive BS included at least one visual or acoustic perception disturbance; cognitive BS included thought interference, blockages, pressure, and perseveration; disturbances of receptive and expressive speech, of abstract thinking, or of discriminating between ideas and perceptions; captivation of attention by details of the visual field; inability to divide attention; unstable ideas of reference; and derealization. Because the SPI-CY requires a minimum age of 13 years for the assessment of three of the 14 BS included in COPER and COGDIS (Online Resource 1), main analyses were conducted on the 11 BS assessed across all age groups to avoid a negative selection bias in the youngest age group.

Furthermore, the positive items of the Structured Interview for Psychosis-Risk Syndromes (SIPS) [23] were assessed; and participants (or a parent, in those of age 8–15) were asked about first or second degree family members with mental problems, treated or untreated, and the diagnosis or, if unknown or never seeking help, a description of these problems (and treatment, if applicable).

Symptom-independent current global level of psychosocial functioning was estimated using the Social and Occupational Functioning Assessment Scale (SOFAS); a score ≤ 70 was considered indicative of low, i.e., clinically significant, impairment in functioning. The Mini-International Neuropsychiatric Interview [24] and its children’s version [25] were used to assess current mental disorders according to DSM-IV criteria.

In both the BEAR and the BEARS-Kid study, interviewers (all clinical psychologists) received intensive 3-month training, especially in the semi-structured context-dependent personalized assessment of psychosis-risk symptoms and mental disorders, to achieve a ≥ 95% concordance rate with the trainers (F.S.-L. and C.M.) before the conduction of interviews. In line with clinical assessments, this routinely included gathering thorough information on:

- novelty/first recognition of the respective disturbance
- situations in which the phenomenon had occurred,
- the degree of externalization/conviction (in case of APS),
- participant’s reaction in response to/explanation of the potential symptom incl. distress (self-perception as a disturbance in own mental processes as an obligate criterion for the rating of BS),
- reactions of others (in case of APS; in particular, others’ opinion on potential “unusual thought content” to control for ‘normal’ subcultural believes),
- potential associations with substance use, somatic/known neurological conditions or hypnagogic/hypnopompic states.

Additionally, weekly supervisions of all symptom ratings performed by FSL or CM ensured excellent, reliable data quality prior to data entry.

### Statistical analysis

Using SPSS 21.0., frequencies were compared by Chi square test, and non-normally distributed interval and ordinal data were evaluated by Mann–Whitney test. In accordance with other studies of age effects in APS [8, 9, 11], logistic regression analyses were used to calculate (I) effects of age groups (8–12; 13–15; 16–17; 18–19; 20–24; 25–29; 30–40) on prevalence rates of BS and their novelty and frequency requirements, and (II) effects of age, BS and BS criteria requirements, and their interaction with age on low psychosocial functioning, and the presence of at least one axis-I disorder as dependent variables. To test (I), we used the enter method and, again in accordance with previous studies [8, 9], the age group with a peak onset of first-episode psychosis (20–24 years) as a reference group. Additionally, bootstrapping was performed to test the reliability of results. To test (II), we employed stepwise logistic regression analyses using both the backward and the forward selection to control for the different suppressor effects associated with each selection mode and, thus, ensure stability of results. In each analysis, the respective BS, age and the “BS × age” interaction term entered as predictors to test the interaction effect against the simple effects. Age rather than age group was entered in these analyses because of the expected small numbers of low functioning and mental disorders per age group. A “BS × age” interaction was only considered as relevant and inspected for its direction by interaction graphs when selected as a significant predictor in both the forward and backward selection. Since bootstrapping cannot be performed with the stepwise regression analysis in SPSS, we subsequently tested the reliability of relevant “BS × age” interactions using bootstrapping of the enter method in regression analysis. Throughout, goodness-of-fit was estimated by the Omnibus test.

For the final sensitivity analyses, stepwise regression analyses were repeated separately for age groups below and above the suggested age thresholds to test if these thresholds could fully account for age effects, in which case age effects should be missing within the age subsamples above and below the age threshold, respectively.

## Results

### Prevalence of BS and age

**Fig. 1:**
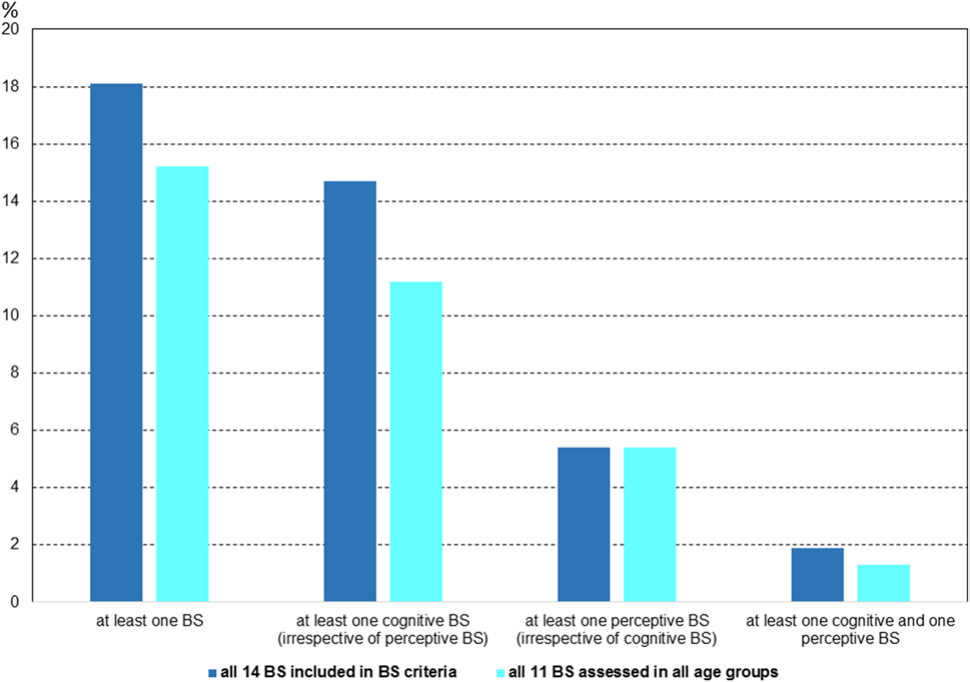
Prevalence rates of basic symptoms (BS) in the whole sample (*N* = 689)

At least one of the 14 BS and at least one of the 11 BS assessed in all age groups were reported by 125 participants (18.1%) and 105 participants (15.2%), respectively, within the 3 months prior to the interview (Fig. 1). Report of at least one BS was unrelated to gender, nationality, parental education, family history of psychosis, and low functioning, but was related to age and other psychopathology, i.e., presence of axis-I disorder or APS (Table 1). COPER criteria were met by 23 participants (3.3%); of these, eight (1.2%) also met the COGDIS criteria.

**Table 1:** Sociodemographic characteristics and prevalence of at least any one basic symptom (BS), irrespective of BS criteria’s frequency and novelty requirements

Characteristic	Subjects with ≥ 1 of 11 BS (*n* = 105)		Subjects without any of 11 BS (*n* = 584)		Statistics^b^				Subjects with ≥ 1 of 14 BS^a^ (*n* = 125)		Subjects without any of 14 BS (*n* = 564)		Total sample (*N* = 689)		Statistics^b^			
	Mdn	*Q*	Mdn	*Q*	*U*		*p*	ES	Mdn	*Q*	Mdn	*Q*	Mdn	*Q*	*U*		*p*	ES
Age	19.5	16.6–26.9	24.1	19.3–29.7	22459.0		< 0.001	0.166	19.8	16.6–26.9	24.3	19.4–29.8	23.3	18.5–29.5	26116.0		< 0.001	0.173
Highest ISCED score of parents	4	3–5	3	3–5	28033.0		0.139	0.056	4	3–5	3	3–5	3	3–5	33242.0		0.297	0.040

	*N*	%	*N*	%	*χ*^2^	*df*	*p*	ES	*N*	%	*N*	%	*N*	%	*χ*^2^	*df*	*p*	ES
Age group (years)																		
8–12	12	26.7	33	73.3	33.335	6	< 0.001	0.220	^c^		^c^		45	6.5	22.738	6	< 0.001	0.221
13–15	11	35.5	20	64.5					13	41.9^g^	18	58.1^g^	31	4.5				
16–17	15	19.2	63	80.8					19	24.4^g^	59	75.6^g^	78	11.3				
18–19	21	25.9	60	74.1					23	28.4^g^	58	71.6^g^	81	11.8				
20–24	18	11.6	137	88.4					25	16.1^g^	130	83.9^g^	155	22.5				
25–29	10	6.9	134	93.1					12	8.3^g^	132	91.7^g^	144	20.9				
30–40	18	11.6	137	88.4					21	13.4^g^	134	86.5^g^	155	22.5				
Male	36	34.3	260	44.5	3.805	1	0.051	0.074	48	38.4	248	44.0	296	43.0	1.296	1	0.255	0.043
Swiss nationality	97	92.4	537	92.0	0.022	1	0.881	0.006	114	91.2	520	92.2	634	92.0	0.139	1	0.709	0.014
≥ 1 current axis-I diagnosis^d^	28	26.7	64	11.0	18.980	1	< 0.001	0.166	31	24.8	61	10.8	92	13.4	17.296	1	< 0.001	0.158
First degree relative with psychosis	1	1.0	5	0.9	0.009	1	0.923	0.004	1	0.8	5	0.9	6	0.9	0.008	1	0.931	0.003
SOFAS ≤ 70^e^	7	6.7	18	3.1	3.270	1	0.071	0.069	7	5.6	18	3.2	25	3.6	1.697	1	0.193	0.050
≥ 1 APS^f^	25	23.8	43	7.4	27.063	1	< 0.001	0.198	30	24.0	38	6.7	68	9.9	34.278	1	< 0.001	0.223

*ISCED* International Standard Classification of Education (http://uis.unesco.org/en/topic/international-standard-classification-education-isced)

^a^Including the three cognitive BS (thought blockages, disturbances of abstract thinking, and derealization) that are only assessable from age 13 onwards

^b^Descriptive statistics: Chi square test for categorical variables with Cramer’s *V* as effect size (ES), Mann–Whitney *U* test for non-normally distributed continuous variables with Rosenthal’s *r* as effect size (ES)

^c^Three cognitive BS (thought blockages, disturbances of abstract thinking, and derealization) are only assessable from age 13 onwards, thus only 11 BS were assessed in this age group

^d^DSM-IV diagnoses: *n* = 57 (8.3%) affective disorders, mainly depression (*n* = 28, 4.1%); *n* = 34 (4.9%) anxiety disorders; *n* = 6 (0.9%) obsessive–compulsive disorders; *n* = 13 (1.9%) eating disorders; *n* = 9 (1.3%) somatization disorders; *n* = 1 (0.8%) exclusively fulfilled criteria of attention deficit/hyperactivity disorder and conduct disorder, respectively, that were only assessed in participants of the BEARS-Kid study (*n* = 119) and not included in the summary variable

^e^SOFAS = Social and Occupational Functioning Scale a value of ≤ 70 indicates presence of at least “some difficulty in social, occupational, or school functioning”

^f^APS = attenuated psychotic symptom according to the Structured Interview for Psychosis-Risk Syndromes (SIPS [21]), no brief intermittent psychotic symptoms reported in sample [7]

^g^% of age group not of BS group sample

Using 20- to 24-year-olds as the reference group, logistic regression analyses indicated a pattern of differential age thresholds for cognitive and perceptive BS. Perceptive BS, especially when occurring at least once in a week (= frequency requirement), were more frequent below age 18. Cognitive BS and COGDIS decreased in the early twenties (Table 2 and Online Resource 2). Omnibus tests revealed acceptable goodness-of-fit for 12 of 15 models; only the three models using presence and novelty requirements of perceptive BS and the frequency requirement of at least one of the 11 BS assessed across all age groups remained insignificant. Although models for the infrequent COPER and COGDIS also possessed sufficient goodness-of-fit, age groups remained largely insignificant (Table 3).

**Table 2:** Prevalence and effects of age on the report of the 11 criteria-relevant basic symptoms (BS) assessed in all age groups, irrespective of novelty and frequency requirements; binary logistic regression analyses with method “enter” and 20- to 24-year-olds as the reference age group

Age group (years)	*β*	SE	Wald (*df* = 1)	*p* after bootstrap	Exp (*β*)	95% CI	Number present	% present
≥ 1 BS (in 20–24 years: *n* = 18, 12%)								
8–12	**1.018**	**0.420**	**5.872**	**0.021**	**2.768**	**1.22–6.31**	**12**	**27**
13–15	**1.432**	**0.451**	**10.061**	**0.001**	**4.186**	**1.73–10.14**	**11**	**36**
16–17	0.595	0.381	2.431	0.113	1.812	0.86–3.83	15	19
18–19	**0.980**	**0.357**	**7.551**	**0.006**	**2.664**	**1.32–5.36**	**21**	**26**
25–29^a^	− 0.566	0.413	1.879	0.156	0.568	0.25–1.28	10	7
30–40	0.000	0.355	0.000	0.999	1.000	0.50–2.00	18	12
≥ 1 cognitive BS (in 20–24 years: *n* = 12, 8%)								
8–12	**0.946**	**0.492**	**3.696**	**0.049**	**2.577**	**0.98–6.76**	**8**	**18**
13–15	**1.584**	**0.497**	**10.164**	**0.002**	**4.875**	**1.84–12.91**	**9**	**29**
16–17^b^	0.671	0.443	2.296	0.116	1.956	0.82–4.66	11	14
18–19	**1.225**	**0.402**	**9.280**	**0.001**	**3.405**	**1.55–7.49**	**18**	**22**
25–29	− 0.496	0.490	1.024	0.299	0.233	0.23–1.59	7	5
30–40	0.000	0.425	0.000	1.000	0.435	0.44–2.30	12	8
≥ 1 perceptive BS (in 20–24 years: *n* = 7, 5%)								
8–12	***0.972***	***0.612***	***2.521***	***0.094***	***2.643***	***0.80–8.77***	***5***	***11***
13–15	0.818	0.720	1.289	0.255	2.265	0.55–9.29	3	10
16–17	***0.882***	***0.537***	***2.694***	***0.088***	***2.416***	***0.84–6.93***	***8***	***10***
18–19	0.094	0.642	0.021	0.8674	1.098	0.31–3.87	4	5
25–29	− 0.504	0.638	0.625	0.431	0.604	0.17–2.11	4	3
30–40	− 0.161	0.568	0.080	0.765	0.851	0.28–2.59	6	4

Significant predictors (*p* < 0.05) are in bold type, predictors with significance at statistical trend (*p* < 0.10) in bold italics

^a^Lower prevalence in comparison to 20- to 24-year-olds became significant when the cognitive BS that are only assessable from age 13 onwards were considered (Exp (*β*) = 0.473; 95% CI 0.23–0.98, *p* (bootstrap) = 0.040)

^b^Higher prevalence in comparison to 20- to 24-year-olds reached statistical trend level when the cognitive BS that are only assessable from age 13 onwards were considered (Exp (*β*) = 1.847; 95% CI 0.89–3.83, *p* (bootstrap) = 0.083)

**Table 3:** Effect of age on presence of basic symptom (BS) criteria

Age group (years)	*β*	SE	Wald (*df* = 1)	*p* after bootstrap	Exp (*β*)	95% CI	*N* present	% present
Cognitive-perceptive BS (COPER)								
8–12^a^	− 17.991	5991.614	0	0.001	0	*n* = 0	0	
13–15	0.979	0.736	1.765	0.137	2.661	0.63–11.27	3	10
16–17	0.294	0.661	0.198	0.646	1.342	0.37–4.90	4	5
18–19	0.491	0.622	0.623	0.432	1.634	0.48–5.53	5	6
25–29	− ***1.751***	***1.086***	***2.596***	***0.065***	***0.174***	***0.02–1.46***	***1***	***1***
30–40	− 0.419	0.656	0.408	0.503	0.658	0.18–2.38	4	3
Cognitive disturbances (COGDIS)								
8–12^a^	− 16.866	5991.614	0	0.001	0	*n* = 0	0	
13–15	**1.663**	**1.020**	**2.657**	**0.010**	**5.276**	**0.71–38.97**	**2**	**7**
16–17	− 16.866	4550.958	0	0.001	0	*n* = 0	0	
18–19	1.079	0.923	1.366	0.106	2.942	0.48–17.98	3	4
25–29	− 16.866	3349.414	0	0.001	0	*n* = 0	0	
30–40	− 0.700	1.230	0.324	0.249	0.497	0.05–5.54	1	1

Binary logistic regression analysis with method “enter” and 20- to 24-year-olds as the reference age group

Significant predictors (*p* < 0.05) are in bold type, predictors with significance at statistical trend (*p* < 0.10) in bold italics

*N* (%) in reference group of 20- to 24-year-olds: COPER: *n* = 6, 4%; COGDIS: *n* = 2, 1%

^a^Both BS criteria include two cognitive BS (thought blockages and derealisation included in COPER; and thought blockages and disturbances of abstract thinking included in COGDIS) that are only assessable from age 13 onwards, thus biasing both BS criteria towards lower frequencies and, consequently, non-significant results in the 8- to 12-year-olds

### Clinical significance of BS and age

Logistic regressions with low functioning as the dependent variable indicated that the interactions of the 11 BS assessed across all age groups with age, rather than the BS parameters themselves, predicted low functioning (Table 4). All selected interaction terms also became significant in univariate logistic regression after bootstrapping. Visual inspection of the interaction graphs (Online Resource 3) showed that, in the presence of the respective BS parameter, low functioning always became more likely with advancing age.

**Table 4:** Effects on low functioning (SOFAS score ≤ 70) of (a) presence of at least one of the basic symptoms (BS) assessed across all age groups as well as of the novelty and frequency requirements of the BS criteria according to the SPI-A/SPI-CY and (b) estimation of ‘interaction with age’ effects

A^a^	*β*	SE	Wald (*df* = 1)	*p* after bootstrap	Exp (*β*)	95% CI	Omnibus test	B^b^	*β*	SE	Wald (*df* = 1)	*p* after bootstrap	Exp (*β*)	95% CI	Omnibus test
*Prevalence of bs irrespective of novelty and frequency requirements*															
≥ 1 BS	Not a significant predictor; only trend level result							≥ 1 BS × age	0.042	0.017	6.265	0.012	1.043	1.01–1.08	$\chi _{{(1)}}^{2}$ = 5.1, *p* = 0.023
≥ 1 cognitive BS	Not a significant predictor							≥ 1 cognitive BS × age	0.039	0.020	3.857	0.050	1.040	1.00–1.08	$\chi _{{(1)}}^{2}$ = 3.1, *p* = 0.077
≥ 1 perceptive BS	Not a significant predictor							≥ 1 perceptive BS × age	No stable model						
*Prevalence of bs meeting novelty irrespective of frequency requirement*															
≥ 1 BS-onset	0.921	0.460	4.015	0.044	2.512	1.02–6.19	$\chi _{{(1)}}^{2}$ = 3.5, *p* = 0.061	≥ 1 BS-onset × age	0.051	0.018	8.414	0.004	1.053	1.02–1.09	$\chi _{{(1)}}^{2}$ = 6.8, *p* = 0.009
≥ 1 cognitive BS-onset	Not a significant predictor; only trend level result							≥ 1 cognitive BS-onset × age	0.047	0.021	5.174	0.023	1.048	1.01–1.09	$\chi _{{(1)}}^{2}$ = 4.1, *p* = 0.042
≥ 1 perceptive BS-onset	Not a significant predictor							≥ 1 perceptive BS-onset × age	No stable model						
*Prevalence of bs meeting frequency irrespective of novelty requirement*															
≥ 1 BS	2.175	0.514	17.894	0.001	8.801	3.21–24.11	$\chi _{{(1)}}^{2}$ = 13.1, *p* < 0.001	≥ 1 BS-frequency × age	0.105	0.021	24.600	< 0.001	1.111	1.07–1.16	$\chi _{{(1)}}^{2}$ = 19.1, *p* < 0.001
≥ 1 cognitive BS-frequency	2.253	0.557	16.352	0.001	9.515	3.19–28.35	$\chi _{{(1)}}^{2}$ = 11.6, *p* = 0.001	≥ 1 cognitive BS-frequency × age	0.100	0.023	19.161	< 0.001	1.106	1.06–1.16	$\chi _{{(1)}}^{2}$ = 14.2, *p* < 0.001
≥ 1 perceptive BS-frequency	Not a significant predictor							≥ 1 perceptive BS-frequency × age	0.091	0.041	4.933	0.026	1.095	1.01–1.19	$\chi _{{(1)}}^{2}$ = 3.7, *p* = 0.055
*Prevalence of BS criteria*															
COPER	2.486	0.529	22.106	0.001	12.019	4.26–33.88	$\chi _{{(1)}}^{2}$ = 15.9, *p* < 0.001	COPER × age	0.111	0.022	25.539	< 0.001	1.117	1.07–1.17	$\chi _{{(1)}}^{2}$ = 20.7, *p* < 0.001
COGDIS	3.448	0.741	21.646	0.001	31.429	7.36–134.31	$\chi _{{(1)}}^{2}$ = 16.3, *p* < 0.001	COGDIS × age	0.176	0.041	18.802	< 0.001	1.192	1.10–1.29	$\chi _{{(1)}}^{2}$ = 19.2, *p* < 0.001

*SOFAS* Social and Occupational Functioning Assessment Scale, *SPI-A/SPI-CY* Schizophrenia Proneness Instrument, Adult/Child & Youth version

^a^Univariate binary logistic regression analyses with method ‘enter’ and subsequent bootstrapping

^b^Binary logistic regression analyses with method ‘backward’ and ‘forward’ using age (in years), the respective criterion requirement and their interaction term as the independent variables. Only stable models are reported (i.e., both methods revealed significant interaction effects). Otherwise, ‘no stable model’ or ‘no interaction effect’ is stated. Age entered as a continuous variable

Logistic regressions with at least one axis-I disorder as the dependent variable, however, indicated that all BS parameters except the simple report of at least one of the nine cognitive BS assessed across all ages were significant predictors of a current non-psychotic mental disorder (Table 5). The interaction of BS with age became significant mostly when the novelty requirement was met, i.e., when BS were reported as having occurred at some time in the past as a previously unknown or considerably less frequent disturbance (Table 5). All selected interaction terms also became significant in univariate logistic regression after bootstrapping. Again, interaction graphs (Online Resource 4) indicated that, in the presence of the respective BS parameter, mental disorders became more likely with advancing age.

**Table 5:** Effects of the presence of any axis-I disorder according to DSM-IV on the presence of at least one basic symptom (BS) assessed across all age groups as well as of the novelty and frequency requirements of the BS criteria according to the SPI-A/SPI-CY and (b) estimation of ‘interaction with age’ effects

A^a^	*β*	SE	Wald (*df* = 1)	*p* after bootstrap	Exp (*β*)	95% CI	Omnibus test	B^b^	*β*	SE	Wald (*df* = 1)	*p* after bootstrap	Exp (*β*)	95% CI	Omnibus test
*Prevalence of bs irrespective of novelty and frequency requirements*															
≥ 1 BS	1.083	0.257	17.715	0.001	2.955	1.78–4.89	$\chi _{{(1)}}^{2}$ = 16.1, *p* < 0.001	≥ 1 BS × age	0.045	0.010	13.427	< 0.001	1.046	1.03–1.07	$\chi _{{(1)}}^{2}$ = 16.5, *p* < 0.001
≥ 1 cognitive BS	Not a significant predictor; only trend level result							≥ 1 cognitive BS × age	No significant interaction						
≥ 1 perceptive BS	1.724	0.353	23.838	0.001	5.607	2.81–11.20	$\chi _{{(1)}}^{2}$ = 20.9, *p* < 0.001	≥ 1 perceptive BS × age	No stable model						
*Prevalence of bs meeting novelty irrespective of frequency requirement*															
≥ 1 BS-onset	1.225	0.2619	22.082	0.001	3.403	2.04–5.67	$\chi _{{(1)}}^{2}$ = 19.9, *p* < 0.001	≥ 1 BS-onset × age	0.056	0.011	25.2509	< 0.001	1.058	1.04–1.08	$\chi _{{(1)}}^{2}$ = 22.8, *p* < 0.001
≥ 1 cognitive BS-onset	0.652	0.316	4.267	0.034	1.920	1.03–3.56	$\chi _{{(1)}}^{2}$ = 3.88, *p* = 0.049	≥ 1 cognitive BS-onset × age	0.030	0.014	4.569	0.033	1.030	1.00–1.06	$\chi _{{(1)}}^{2}$ = 4.1, *p* = 0.043 ^c^
≥ 1 perceptive BS-onset	1.828	0.360	25.754	0.001	6.219	3.07–12.60	$\chi _{{(1)}}^{2}$ = 22.8, *p* < 0.001	≥ 1 perceptive BS-onset × age	0.090	0.017	26.805	< 0.001	1.094	1.06–1.13	$\chi _{{(1)}}^{2}$ = 26.9, *p* < 0.001
*Prevalence of bs meeting frequency irrespective of novelty requirement*															
≥ 1 BS	1.941	0.391	24.677	0.001	6.946	3.24–14.98	$\chi _{{(1)}}^{2}$ = 21.9, *p* < 0.001	≥ 1 BS-frequency × age	No stable model						
≥ 1 cognitive BS-frequency	1.783	0.494	16.110	0.001	5.945	2.49–14.20	$\chi _{{(1)}}^{2}$ = 14.1, *p* < 0.001	≥ 1 cognitive BS-frequency × age	No stable model						
≥ 1 perceptive BS-frequency	2.116	0.680	9.675	0.001	8.301	2.19–31.50	$\chi _{{(1)}}^{2}$ = 8.9, *p* = 0.003	≥ 1 perceptive BS-frequency × age	0.127	0.043	8.879	0.003	1.136	1.04–1.24	$\chi _{{(1)}}^{2}$ = 10.7, *p* = 0.001
*Prevalence of BS criteria*															
COPER	2.050	0.434	22.340	0.001	7.769	3.32–18.18	$\chi _{{(1)}}^{2}$ = 20.2, *p* < 0.001	COPER × age	No significant interaction						
COGDIS	2.406	0.739	10.595	0.001	11.086	2.60–47.20	$\chi _{{(1)}}^{2}$ = 10.4, *p* = 0.001	COGDIS × age	0.123	0.039	9.790	0.002	1.131	1.05–1.22	$\chi _{{(1)}}^{2}$ = 11.7, *p* = 0.001

*SPI-A/SPI-CY* Schizophrenia Proneness Instrument, Adult/Child & Youth version

^a^Univariate binary logistic regression analyses with method ‘enter’ and subsequent bootstrapping

^b^Binary logistic regression analyses with method ‘backward’ and ‘forward’ using age (in years), the respective criterion requirement and their interaction term as the independent variables. Only stable models are reported (i.e., both methods revealed significant interaction effects). Otherwise, ‘no stable model’ or ‘no interaction effect’ is stated. Age entered as a continuous variable

### Examination of age thresholds

Finally, sensitivity analyses were run on the suggested age thresholds for cognitive and perceptive BS (Online Resources 5 and 6) to examine if an age effect was still detectable within the respective age subgroups. Because the age threshold for cognitive BS ran through the reference group of 20- to 24-year-olds, we tested both “borders”, i.e., age 20 and age 25, to examine a bias towards either age. First, using a 20-year threshold in the nine cognitive BS assessed across all age groups, within the younger age group, only the interaction of age with cognitive BS meeting the frequency requirement of occurrence at least once in a week predicted low functioning and the presence of mental disorder—in the latter case, only in the older age group. Within the older age subgroup, a “cognitive BS × age” effect on low functioning was revealed. Additionally, when all 12 cognitive BS were considered, interaction effects of the 12 cognitive BS meeting the novelty requirements on axis-I disorders and of the presence of the 12 cognitive BS on low functioning became significant in the older age group. These findings did not change when using a 25-year threshold, thus indicating an age threshold for cognitive BS “within the early twenties”.

With regard to the 18-year threshold in perceptive BS, only one within-group age interaction effect with age on low functioning was detected in the older age group, while age effects on mental disorder remained only for the novelty requirement in 18- to 40-year-olds. Within 8- to 17-year-olds, all three interactions with age maintained their significant effect on the presence of a mental disorder, when age entered the model as a second significant predictor. These results indicated that the association between perceptive BS and an axis-I disorder became stronger with advancing age, in particular when perceptive BS were not reported as a trait (i.e., met the novelty requirement) and occurred at least once in a week (i.e., met the frequency requirement).

Overall, the majority of interaction effects that had been significant in the whole sample were not significant any longer after splitting the sample according to the respective age thresholds suggested for cognitive and perceptive BS. Commonly, interactions terms became significant within an age subgroup when the frequency requirement or at least the novelty requirement was met, indicating that the age effect on the association of the sheer prevalence of BS with mental disorder or impaired functioning was predominately related to the respective age threshold. However, in all instances in which interaction terms became significant, they indicated that, in the presence of the respective BS parameter, low functioning and mental disorder, respectively, became more likely with advancing age.

## Discussion

### Prevalence and clinical significance of BS

Within our random, representative community sample of never-psychotic 8- to 40-year-olds, 18.1% reported BS included in either COPER or COGDIS, mainly cognitive BS, in clinical interviews carried out by well-trained clinical psychologists. Only 3.3% met BS criteria, in all cases COPER; 1.2% additionally met criteria for COGDIS, which is part of the clinical high-risk criteria recommended for early detection of psychosis within the guidance project of the European Psychiatric Association [5]. Thus, at least one of the 14 BS included in COPER and COGDIS was reported almost twice as frequently as were APS (9.9%), and COPER was reported about 2.5 times more often than was the APS criterion (1.3%) in the same sample [8]. In contrast, COGDIS was as rare as the APS criterion, and at 4.4%, at least one perceptive BS occurred as infrequently as did at least one perceptive APS (4.9%) [8]. Report of BS was moderately related to report of APS and, though to a lesser degree, to more frequent current non-psychotic DSM-IV axis-I disorders. Furthermore, when reported to occur not as a trait (= novelty requirement) and/or at least weekly (= frequency requirement), BS were related to functional impairment. Thus, as reported for APS [8], our findings indicate some clinical significance of BS at the community level with a stronger relation to subthreshold psychotic symptomatology than to non-psychotic disorders.

### Effects of age on prevalence and clinical significance of BS

As expected from recent reports on APS [7–9], age effects were also revealed in prevalence and clinical significance of BS. Yet, unexpectedly, these did not follow the age threshold in the psychopathological significance of APS around age 15/16 years [7–10]. Compared to APS assessed in the same sample [8], developmental aspects indicated by age seemed to play an even stronger role in BS. APS in the community had shown more direct associations with functional impairment and the presence of a mental disorder and lesser interactions with age [8]. This stronger impact of age, i.e., neurodevelopmental state, on BS is well in line with the proposed strong neurobiological basis of BS [12, 13, 16]. All interactions of BS and age pointed toward an increase in clinical significance (i.e., an increase in their association with the presence of either a mental disorder or functional impairment) with age, while the prevalence rates of BS, both cognitive and perceptive, decreased with age.

With regard to prevalence rates, the infrequency of events, especially in terms of perceptive BS, and the rather small subsample size of some age groups likely constrained the power of age group analyses, despite our large sample. Yet, even with this limitation, regression analyses revealed an age-related pattern in BS prevalence that differed between perceptive and cognitive BS. These prevalence patterns suggested an age threshold for perceptive BS around the turn from late adolescence to young adulthood (i.e., age 18) and one for cognitive BS in young adulthood (i.e., within the early twenties) rather than a general effect of age across all age groups. In particular when BS were reported as occurring infrequently (i.e., did not meet the frequency requirement) but also when they were reported as a trait-like phenomenon (i.e., did not meet the novelty requirement), age effects on the association between BS and either proxy measure of clinical significance (i.e., mental disorder and functional impairment) could only rarely be observed within the age subgroups below and above the respective age threshold. Further, the clinical significance of both cognitive and perceptive BS was lower in the age subgroups below their respective threshold compared to the subgroup above the respective age threshold.

While differences between perceptive and cognitive BS were expected, the emerging age thresholds above the age threshold for APS were unexpected. The BS concept and current models of symptom development in the early phases of psychosis consistently assume that (attenuated) psychotic symptoms follow BS [4, 5, 12, 13]; and first retrospective assessments of prodromal symptoms in patients with first-episode psychosis have broadly supported this view [14]. Consequently, from this assumed earlier onset of BS, we had expected a younger, if different, age threshold compared to the APS threshold of 15/16 years [7–9, 11]. Our results, however, indicate that an earlier onset in the early course of psychosis does not translate into an earlier age threshold in prevalence and clinical significance.

### An integrated developmental model of psychosis-risk symptoms

**Fig. 2:**
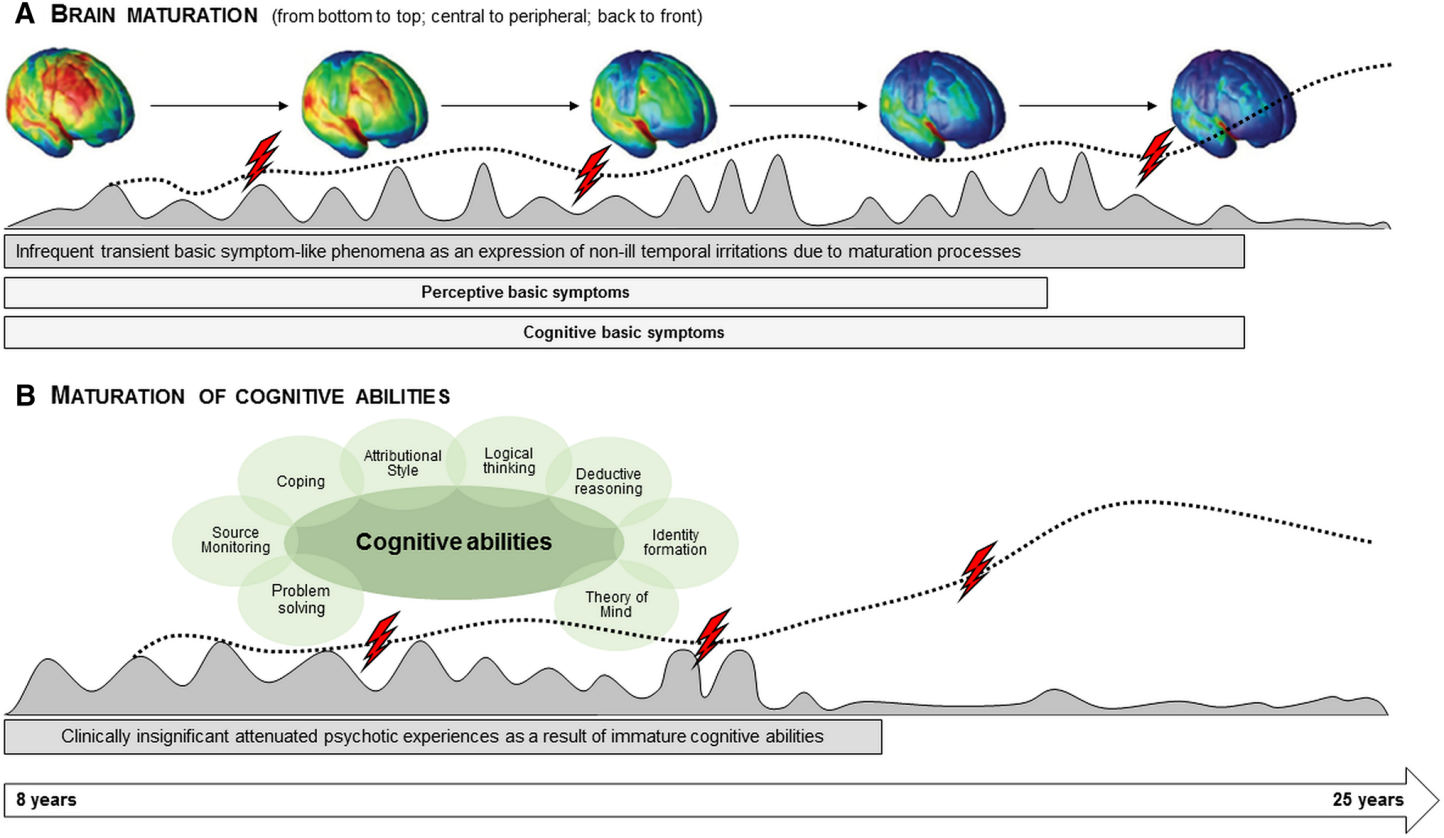
Illustration of the possible relationship between basic symptoms and brain maturation, and between attenuated psychotic symptoms and maturation of cognitive abilities. This model assumes that **A** subtle subclinical disturbances in cognitive and perceptive information processing phenomenologically identical to basic symptoms (BS) might occur during childhood and adolescence as infrequent temporary expressions of minor transient dysfunctions in the wake of brain maturation processes (gray-shaded curve). However, if these disturbances, i.e., BS, occur more frequently (i.e., meet the frequency requirements of the BS criteria) and are persistent (dotted line) they might indicate disturbances in brain maturation that, in line with a neurodevelopmental model of psychosis, predispose to the development of psychosis. A genetic predisposition, childhood adversities or other risk factors as well as stressful life-events and cognitions promoting the development of attenuated psychotic symptoms (APS), such as poor coping, externalization biases or poor source monitoring, (risk factors/stressors indicated by flashes) might further fuel the development and persistence of information processing disturbances. On the other hand, the model assumes that **B** unusual perceptual experiences or thought contents identical to APS might occur during childhood and early adolescence as an expression of not yet fully matured cognitive abilities (gray-shaded curve). If their maturation is impaired by risk factors or stressors (flashes) or neurodevelopmental disturbances in information processing (incl. BS), APS might persist or progress (dotted line), potentially leading to schizotypal traits and/or psychosis

A possible explanation of these diverging age thresholds for APS and BS is offered by the BS concept. This assumes BS to be “substrate-close” [12], i.e., to be the most direct expression of the underlying neurobiological aberrations, and APS and psychotic symptoms to be the result of dysfunctional coping with daily hassles and adversities [13]. In light of this, APS would be affected primarily by the development of cognitive abilities, while BS would be affected primarily by brain maturation (Fig. 2).

Brain maturation progresses from bottom to top, central to peripheral, and back to front within the first 2.5 decades of life [26, 27] and, thus, continues into young adulthood, particularly in the frontal regions. Consequently, following the above line of argument, BS-like phenomena likely occur temporarily, infrequently (i.e., below the requirements on frequencies by BS criteria), and randomly as part of major brain maturation processes without being clinically significant and decrease in prevalence once brain maturation has attained the adult corridor. This interpretation fits well with the age threshold of 18 years for perceptive BS, which are likely related to earlier maturing occipitotemporal regions, and an age threshold within the early twenties for cognitive BS, which are likely related to the lastly maturing frontal regions [28, 29] (Fig. 2).

On a cellular level, prefrontal inhibitory synapses strongly increase from age 15 onwards and reach their maximum in the early twenties, while excitatory synapses are eliminated [30, 31]. Thus, as supported by first electrophysiological and imaging studies on COPER/COGDIS samples or using SPI-A total scores, BS might be an expression of an altered excitatory–inhibitory balance [16].

In contrast, APS might be more prevalent but less clinically significant before the development of major cognitive abilities [32]. The BS concept [13, 33] and developmental models of APS [34, 35] assume that (attenuated) psychotic symptoms result from dysfunctional coping with first symptoms, e.g., BS and/or stressors, when a vulnerable person’s resilience and protective factors are overstrained (Fig. 2). These dysfunctional coping strategies might include the development of inadequate explanatory models [34]. Thus, dysfunctional coping might be the link between APS and functional impairment, which were found to be moderately related, while BS were only related to functional impairment in the rare event of their at-least-weekly occurrence (Fig. 2).

As a result of the above and in line with a neurodevelopmental model of psychosis [1, 36], the cognitive and perceptive BS included in COPER and COGDIS might signal a risk of developing a psychosis that is potentially related to aberrant brain maturation, e.g., by excessive synaptic pruning [16, 37, 38], when these BS begin in adolescence, occur frequently and persist beyond the respective brain maturation age. In contrast, they might signal psychosis-risk related to neurodegenerative mechanisms, such as neuroinflammation or oxidative stress [39, 40], when they start or re-emerge after brain maturation is complete (Fig. 2). Should this view be supported in future, it will not only give important new insight into the pathogenesis of psychosis, but will also have important therapeutic implications. It would support the search for both neuroprotective and anti-inflammatory interventions for BS—depending on their course, and the emphasis on cognitive–behavioral interventions for APS. The combination of both interventions [41], however, might be indicated in patients for whom the highest risk for psychosis has already been demonstrated, i.e., help-seekers with both APS and COGDIS [42].

### Strengths and limitations

While our results open exciting perspectives for future early psychosis research, some limitations have to be kept in mind [8]. An obvious limitation refers to the large number of analyses we have performed, which may have increased the risk for a type 1 error. However, it was proposed that no accumulation of the assessment-related type I error occurs in multiple testing when assessments are completely dependent of each other [43]. Thus, with the data on frequency and novelty requirements completely depending on the prevalence data (i.e., a BS can only be rated for novelty and frequency of occurrence when it is reported to be present), an accumulation of the type I error by analyses of the criteria-related requirements in addition to the prevalence data can be assumed to be low if not absent. The same consideration applies to the dependency of the cognitive and perceptive BS subgroup analyses on the total BS analyses, i.e., on the variable “at least one BS”. However, to reduce any remaining type I error accumulation related to the multiple analyses of different outcomes, we applied bootstrapping to validate the accuracy of our results.

Moreover, our results call for replication in clinical samples (as provided already for attenuated and transient psychotic symptoms [9, 11]) as well as in even larger community samples with a similarly broad age range and sufficiently large subsamples below the age of 16. These should be large enough to enable simultaneous study of the interaction between age and gender, since gender differences in brain maturation [44, 45] might lead to lower age thresholds in the clinical significance of BS and possibly APS in females compared to males. Gender might also play a role in higher age, particularly with regard to the second onset peak of schizophrenia in women [46]; and possible gender-related age effects on the prevalence and clinical significance of BS in samples older than age 40 still warrant examination.

Furthermore, more detailed assessment of the age-at-onset and course of BS in future studies will support an estimation of the relative contribution of neurodevelopmental processes with an onset of BS below the respective age threshold and of neurodegenerative processes with an onset of BS above the respective age threshold on the occurrence of BS beyond their progression into frank psychosis. Their longitudinal examination will clarify their relation to frank psychosis, other mental disorders, subclinical states with mental problems, mental health and mental well-being; in addition, resilience and protective factors should be assessed for their assumed high impact on the development of symptoms thought to be based on BS, such as attenuated and frank psychotic symptoms [47].

Future studies on developmental effects should also address the role of BS that are part of the Adynamia section of the SPI-CY (e.g., disturbances in drive and affect and unspecific cognitive BS) and that were suggested to play an important role in the early detection of psychosis in children and adolescents, but not adults [14, 15].

Lastly, differences in assessment might be considered to have affected the results. However, we have found that both face-to-face and telephone-interviews enabled a reliable assessment of APS and BS across age groups [20] and that BS age thresholds did not follow the age threshold of assessment modes. Rather, the use of psychosis-risk criteria identical to those adopted in clinical settings and the assessment of symptoms by an established interview for BS, conducted by trained and closely supervised clinical psychologists, are strengths of this study and ensure the high quality of the data.

### Outlook

In concert with earlier findings of an age effect on APS [7–10], our findings emphasize the urgent need to address the differential effects of perceptive and non-perceptive (i.e., cognitive) psychosis-risk phenomena and their interaction with age in terms of neuro-psychological and neurobiological development in future studies, particularly longitudinal studies of conversion to psychosis [16, 48]. Further, they indicate a need for differential examination of developmental factors affecting prevalence and clinical significance of APS and BS. Such symptomatically sophisticated future studies hold the potential to shed new light on the development of psychosis and the various neuro-psychological and neurobiological processes involved in this, likely to different degrees [49]. Because different processes might signal different treatment needs, such an in-depth understanding of the development of different symptoms will also serve the development of more efficient personalized interventions.

## Electronic supplementary material

Below is the link to the electronic supplementary material.

Supplementary material 1 (DOCX 547 KB)
