# Supplementary material for: Age effects on basic symptoms in the community: A route to gain new insight into the neurodevelopment of psychosis?
Source: Eur Arch Psychiatry Clin Neurosci. 2018 Oct 25;270(3):311–24. doi: 10.1007/s00406-018-0949-4 (PMC7069926; doi:10.1007/s00406-018-0949-4)

**European Archives of Psychiatry and Clinical Neuroscience**

**Supplementary Material**

**to**

**Age effects on basic symptoms in the community: A route to gain new insight into the neurodevelopment of psychosis?**

Frauke Schultze-Lutter^1,2 † *^, Stephan Ruhrmann^3 *^, Chantal Michel^1^, Jochen Kindler^1^, Benno G. Schimmelmann^1,4 *^, Stefanie J. Schmidt^1,3,5 *¶^

* shared first/senior authorship

† corresponding author

^1^ University Hospital of Child and Adolescent Psychiatry and Psychotherapy, University of Bern, Bern, Switzerland

^2^ Department of Psychiatry and Psychotherapy, Medical Faculty, Heinrich-Heine-University, Düsseldorf, Germany

^3^ Department of Psychiatry and Psychotherapy, University of Cologne, Cologne, Germany

^4^ University Hospital of Child and Adolescent Psychiatry, University Hospital Hamburg-Eppendorf, Hamburg, Germany

^5^ Department of Clinical Psychology and Psychotherapy, University of Bern, Bern, Switzerland

**Corresponding author**

Department of Psychiatry and Psychotherapy,

Medical Faculty, Heinrich-Heine-University,

Bergische Landstrasse 2

40629 Düsseldorf, Germany

E-mail: [frauke.schultze-lutter@](mailto:frauke.schultze-lutter@)lvr.de

Phone: +49 (0)221 922 2029

**Online Resource 1** Basic symptom (BS) criteria, and rough definitions of BS

**Cognitive-Perceptive Basic Symptoms (COPER)**

At least any 1 of the following 10 BS that is reported as a disruption in a person’s “normal” self (= novelty requirement) with a SPI-A / SPI-CY score of ≥3 (i.e., at least weekly occurrence = frequency requirement) within the last 3 months ***and*** first occurrence ≥12 months ago (= onset requirement):

- ***Thought interference***: Irrelevant, emotionally neutral thoughts with no special meaning and no association with the intended thought are intruding on and disturbing the young person’s train of thought, without it being lost.
- ***Thought perseveration***: A kind of thought interference in that intruding emotionally neutral and irrelevant thoughts or images occur not just once but repeatedly.
- ***Thought pressure***: A self-reported ‘chaos’ of thoughts in that successively occurring thoughts are not linked by any common thread, and are completely unrelated to each other or to the young person’s intended line of thought.
- ***Thought blockages (assessable from age 13)***: Sudden interruption in the flow of thoughts, or experiences of the mind suddenly going blank, of a fading (slipping) of thoughts or of losing the thread of thoughts, with the original topic being recalled subsequently or lost completely.
- ***Disturbance of receptive speech***: A disturbance in the understanding of simple everyday words. When reading or listening to others, the young person struggles to comprehend the meaning of words, word sequences or sentences, even if the young person concentrates on the text or speech and has perceived it accurately.
- ***Decreased ability to discriminate between ideas and perception, fantasy and true memories***: A self-recognized difficulty in locating the source of an experience/memory (external vs. internal mental) that results in an inability to immediately distinguish between imaginations and perception, or pure fantasy and true memories.
- ***Unstable ideas of reference***: Subjective, subclinical experiences of self-reference for that no explanation outside own mental processes are sought, and that is immediately overcome.
- ***Derealisation (assessable from age 13)***: A change in how one relates emotionally to the environment, which is experienced commonly as an estrangement and detachment from the visual world, or rarely as an increased emotional affinity for the environment.
- ***Visual perception disturbances*** (excl. blurred vision and hypersensitivity to light): Misperceptions of aspects of the visual field while the young person is fully aware of their true appearance and, therefore, attributes his or her misperception to a problem with eyesight or mental processes.
- ***Acoustic perception disturbances*** (excl. hypersensitivity to sounds/noises): Misperceptions of acoustic stimuli while the young person is fully aware of the true sound and, therefore, tends to attribute his or her misperception to a problem with hearing or mental processes.

**Cognitive Disturbances (COGDIS)**

At least any 2 of the following 9 BS that is reported as a disruption in a person’s “normal” self (= novelty requirement) with a SPI-A / SPI-CY score of ≥ 3 (i.e., at least weekly occurrence = frequency requirement) within the last 3 months:

- ***Inability to divide attention***: A difficulty in dealing with demands that involve more than one sensory modality at a time and thus does not concern demands that would require quick switching of attention.
- ***Captivation of attention by details of the visual field***: Domination of the visual field by a random single aspect of it that captures the young person’s whole attention, impedes paying attention to other aspects and causes difficulties in turning away from it.
- ***Thought interference*** (see COPER)
- ***Thought pressure*** (see COPER)
- ***Thought blockages*** (see COPER) ***(assessable from age 13)***
- ***Disturbance of receptive speech*** (see COPER)
- ***Disturbance of expressive speech***: A subjective difficulty in verbal fluency and clarity of expression, with words required to express simple ideas being not forthcoming or delayed.
- ***Unstable ideas of reference*** (see COPER)
- ***Disturbances of abstract thinking (assessable from age 13)***: Deficits in the comprehension of any kind of abstract, figurative or symbolic phrases or content, as well as the phenomena of ‘concretism’ (a limitation of the ability to go beyond the literal meaning of words, sentences or phrases).

A general requirement of BS is their novelty, i.e., their report as a disruption in a person’s “normal” self. Self-recognized aberrations in mental processes that have always been present in a trait-like manner can be rated in SPI-A / SPI-CY (rating of “7”) but are not accounted for as BS in the strict sense and, consequently, would not contribute to BS criteria.

More in-depth definitions of BS as well as example statements of patients and example questions for their assessment are provided in the SPI-A / SPI-CY, orderable at [www.fioriti.it.](http://www.fioriti.it/)

**Online Resource 2.** Prevalence and effects of age on the report of the 11 criteria-relevant basic symptoms (BS) assessed in all age groups, when meeting the requirement for novelty (not always present in same frequency) and frequency (experienced at least once in a week within past three months), respectively; binary logistic regression analyses with method “enter” and 20- to 24-year-olds as the reference age group.

|  |  | **4a) PREVALENCE OF BS MEETING NOVELTY REQUIREMENT** | | | | | | | | **4b) PREVALENCE OF BS MEETING FREQUENCY REQUIREMENT** | | | | | | | |
| --- | --- | --- | --- | --- | --- | --- | --- | --- | --- | --- | --- | --- | --- | --- | --- | --- | --- |
|  | Age group | β | SE | Wald (df=1) | p after bootstrap | Exp (β) | 95% CI | Number present | % present | β | SE | Wald (df=1) | p after bootstrap | Exp (β) | 95% CI | Number present | % present |
| ≥1 BS * | ***8-12 yrs.*** | ***0.777*** | ***0.438*** | ***3.153*** | ***0.069*** | ***2.175*** | ***0.92-5.13*** | ***10*** | ***22*** | 0.144 | 0.835 | 0.030 | 0.707 | 1.155 | 0.23-5.93 | 2 | 4 |
|  | **13-15 yrs.** | **1.288** | **0.459** | **7.878** | **0.004** | **3.624** | **1.48-8.91** | **10** | **32** | **1.303** | **0.679** | **3.686** | **0.035** | **3.679** | **0.97-13.91** | **4** | **13** |
|  | 16-17 yrs. | 0.510 | 0.387 | 1.734 | 0.174 | 1.664 | 0.78-3.56 | 14 | 18 | 0.294 | 0.661 | 0.198 | 0.612 | 1.342 | 0.37-4.90 | 4 | 5 |
|  | **18-19 yrs.** | **0.980** | **0.357** | **7.551** | **0.002** | **2.664** | **1.32-5.36** | **21** | **26** | ***0.854*** | ***0.574*** | ***2.212*** | ***0.096*** | ***2.349*** | ***0.76-7.24*** | ***7*** | ***9*** |
|  | 25-29 yrs. ^#^ | -0.566 | 0.431 | 1.879 | 0.176 | 0.568 | 0.25-1.28 | 10 | 7 | -1.050 | 0.825 | 1.622 | 0.107 | 0.350 | 0.07-1.76 | 2 | 1 |
|  | 30-40 yrs. | -0.361 | 0.383 | 0.889 | 0.377 | 0.697 | 0.33-1.48 | 14 | 8 | -0.419 | 0.656 | 0.408 | 0.510 | 0.658 | 0.18-1.38 | 4 | 3 |
| ≥1 cognitive BS * | 8-12 yrs. | 0.606 | 0.532 | 1.300 | 0.228 | 1.833 | 0.65-5.20 | 6 | 13 | -17.99 | 5991.6 | 0.000 | 0.001 | 0.000 | 0.00- | 0 |  |
|  | **13-15 yrs.** | **1.422** | **0.509** | **7.812** | **0.006** | **4.145** | **1.53-11.23** | **8** | **26** | 0.979 | 0.736 | 1.765 | 0.135 | 2.661 | 0.63-11.27 | 3 | 10 |
|  | 16-17 yrs. | 0.561 | 0.453 | 1.535 | 0.204 | 1.752 | 0.72-4.26 | 10 | 13 | -1.132 | 1,089 | 1,079 | 0.150 | 0.323 | 0.04-2.73 | 1 | 1 |
|  | **18-19 yrs.** | **1.225** | **0.402** | **9.280** | **0.002** | **3.405** | **1.55-7.49** | **18** | **22** | 0.854 | 0.574 | 2.212 | 0.126 | 2.349 | 0.76-7.24 | 7 | 9 |
|  | 25-29 yrs. ^#^ | -0.496 | 0.490 | 1.024 | 0.324 | 0.609 | 0.23-1.59 | 7 | 5 | -1.050 | 0.825 | 1.622 | 0.114 | 0.350 | 0.07-1.76 | 2 | 1 |
|  | 30-40 yrs. | -0.308 | 0.456 | 0.457 | 0.507 | 0.735 | 0.30-1.80 | 9 | 6 | -0.713 | 0.716 | 0.991 | 0.254 | 0.490 | 0.12-2.00 | 3 | 2 |
| ≥1 perceptive BS * | **8-12 yrs.** | ***0.972*** | ***0.612*** | ***2.521*** | ***0.087*** | ***2.643*** | ***0.80-8.77*** | ***5*** | ***11*** | **1.969** | **1.237** | **2.534** | **0.011** | **7.163** | **0.63-80.87** | **2** | **4** |
|  | **13-15 yrs.** | 0.818 | 0.720 | 1.289 | 0.203 | 2.265 | 0.55-9.29 | 3 | 10 | **1.636** | **1.428** | **1.312** | **0.013** | **5.133** | **0.31-84.35** | **1** | **3** |
|  | **16-17 yrs.** | ***0.882*** | ***0.537*** | ***2.694*** | ***0.081*** | ***2.416*** | ***0.84-6.93*** | ***8*** | ***10*** | **2.119** | **1.127** | **3.536** | **0.017** | **8.324** | **0.91-75.79** | **4** | **5** |
|  | 18-19 yrs. | 0.094 | 0.642 | 0.021 | 0.857 | 1.098 | 0.31-3.87 | 4 | 5 | -16.17 | 4465.9 | 0 | 0.001 | 0.000 | 0.00- | 0 |  |
|  | 25-29 yrs. | -0.504 | 0.638 | 0.625 | 0.412 | 0.604 | 0.17-2.11 | 4 | 3 | -16.17 | 3349.4 | 0 | 0.001 | 0.000 | 0.00- | 0 |  |
|  | 30-40 yrs. | -0.580 | 0.637 | 0.827 | 0.343 | 0.560 | 0.16-1.95 | 4 | 3 | 0 | 1.419 | 0 | 0.997 | 1.000 | 0.06-16.13 | 1 | 1 |

Significant predictors (p<0.05) are in bold type, predictors with significance at statistical trend (p<0.10) in bold Italics.

*N (%) in reference group of 20- to 24-year-olds: ≥1 BS meeting novelty requirement: n=18, 12%; ≥1 BS meeting frequency requirement: n=6, 4%; ≥1 cognitive BS meeting novelty requirement: n=12, 8%; ≥1 cognitive BS meeting frequency requirement: n=6,4 %; ≥1 perceptive BS meeting novelty requirement: n=7, 5%; ≥1 perceptive BS meeting frequency requirement: n=1, 1%.

^#^ Lower prevalence in comparison to 20- to 24-year-olds became significant when the cognitive BS that are only assessable from age 13 onwards were considered for any BS meeting novelty requirement (Exp(β)=0.473; 95%-CI: 0.23-0.98, p(bootstrap)=0.046), and for any cognitive BS meeting frequency requirement (Exp(β)=0.345; 95%-CI: 0.09-1.30, p(bootstrap)=0.058).

**Online Resource 3** Interactions of age and presence of ≥1 basic symptom (BS) parameter significantly impacting on impaired psychosocial functioning (score ≤70 on the Social and Occupational Functioning Assessment Scale, SOFAS) as proxy measure of clinical significance according to Table 4

Please note: Important German terms and unlabeled variable names are translated and explained, respectively, at first occurrence in the document. For immediate recognition, they are given in red.


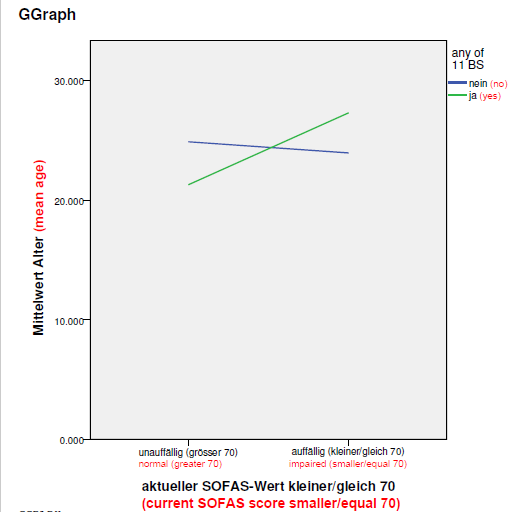

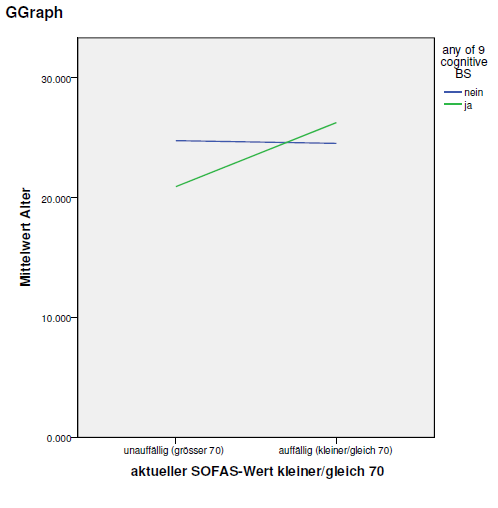

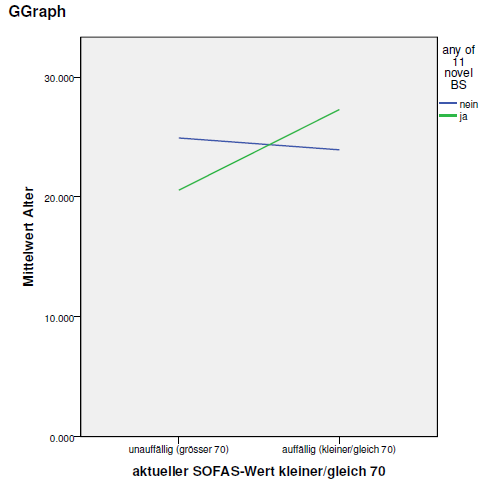


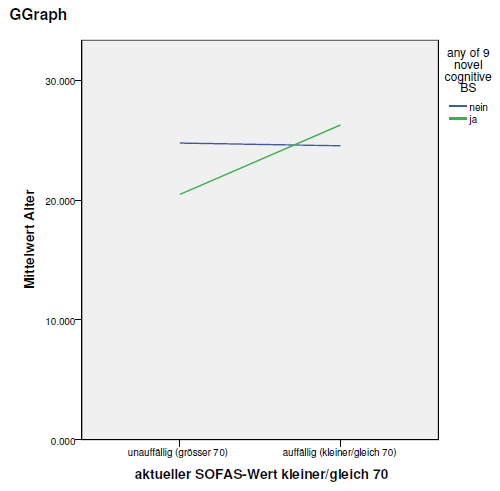

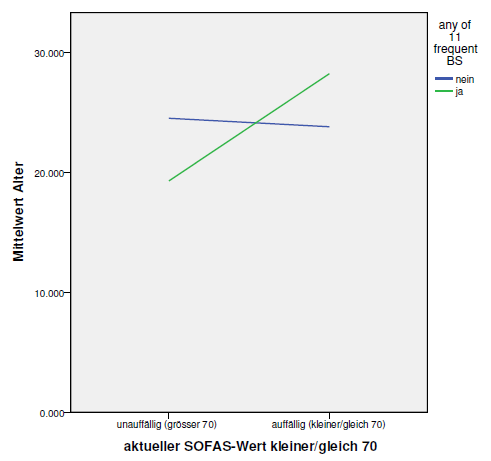

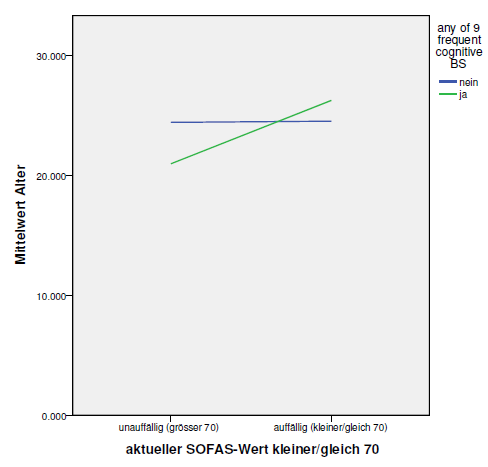


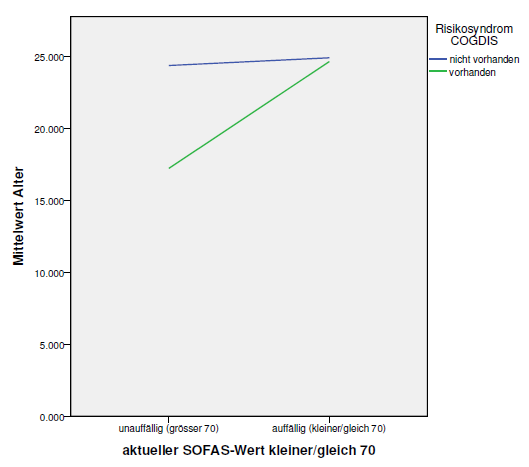

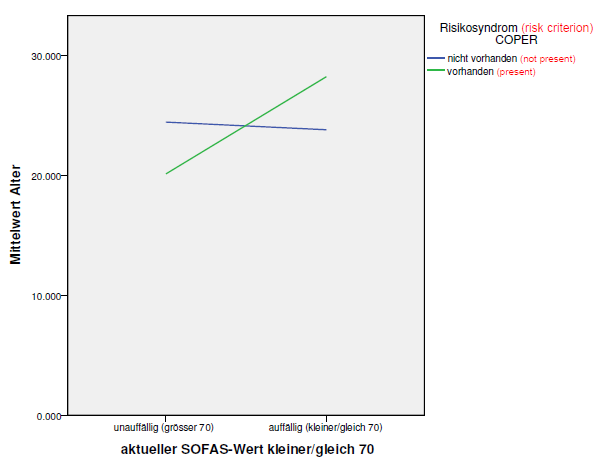

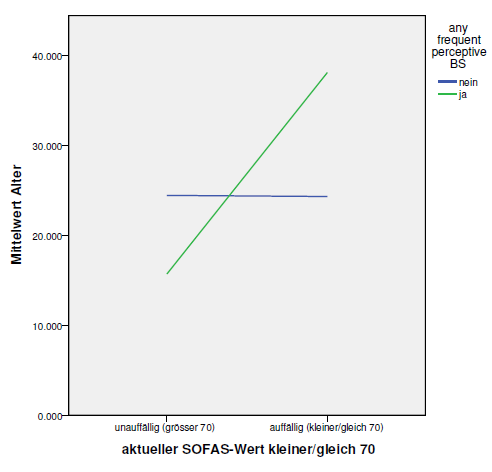


**Online Resource 4** Interactions of age and presence of ≥1 basic symptom (BS) parameter significantly impacting on presence of any nonpsychotic axis-I disorder according to DSM-IV as a proxy measure of clinical significance according to Table 5


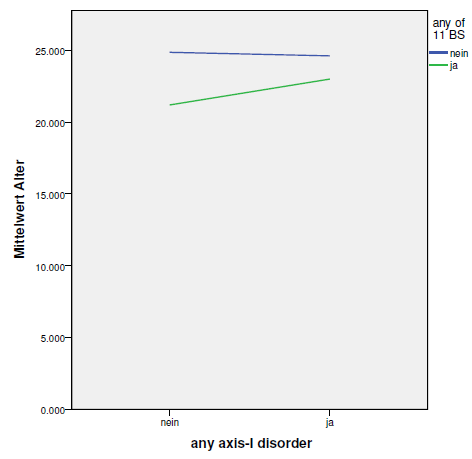

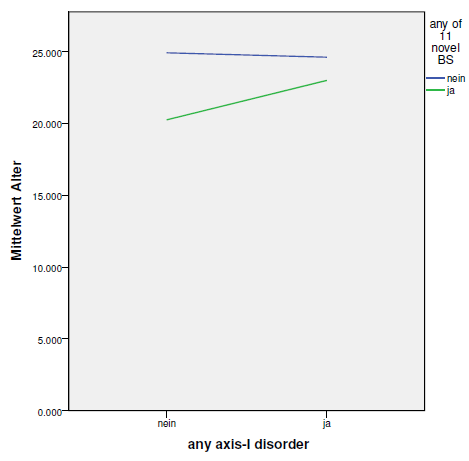

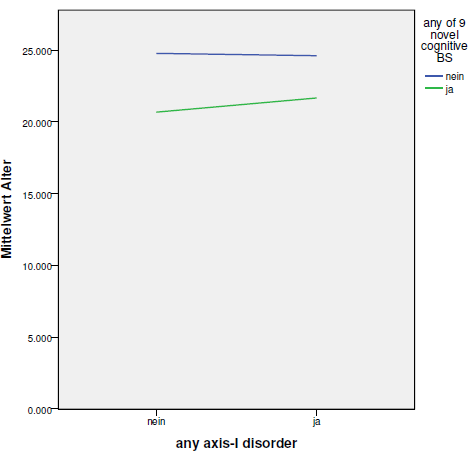


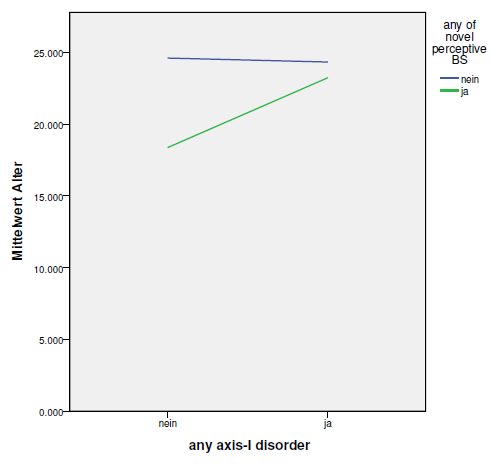

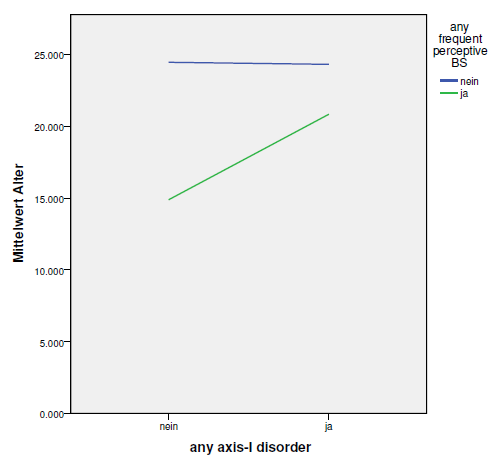


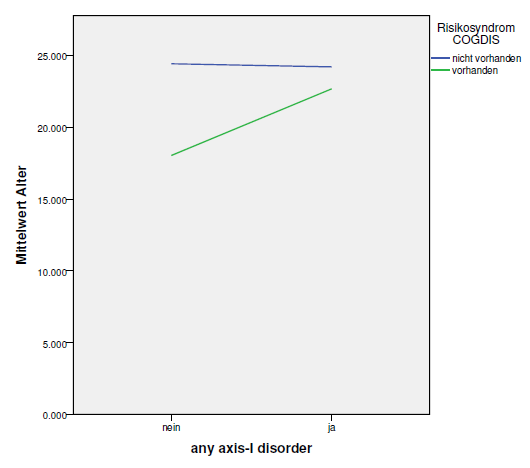


**Online Resource 5** Sensitivity analyses of effects of the interaction of age with the cognitive and perceptive basic symptoms (BS) and their novelty and frequency requirements on presence of any axis-I disorder according to DSM-IV and low functioning (SOFAS score ≤ 70) within the age groups divided by a 20-year age threshold and a 18-year age threshold, respectively

| **Cognitive basic symptoms (BS)** (9 BS assessed across all age groups) | | | | | | | | | | | | | | | | | | | | | | | | | | | | | | |
| --- | --- | --- | --- | --- | --- | --- | --- | --- | --- | --- | --- | --- | --- | --- | --- | --- | --- | --- | --- | --- | --- | --- | --- | --- | --- | --- | --- | --- | --- | --- |
|  | β | SE | | Wald (df=1) | | p after bootstrap | | Exp (β) | | | 95% CI | | Omnibus Test |  | | β | SE | | | | Wald (df=1) | | | p after bootstrap | Exp (β) | 95% CI | | Omnibus Test | | |
| **EFFECT ON PRESENCE OF AXIS-I DISORDER** | | | | | | | | | | | | | | | | | | | | | | | | | | | | | | |
| Age group 8-19 years | | | | | | | | | | | | | | Age group 20-40 years | | | | | | | | | | | | | | | | |
| *≥1 cognitive BS × ageb* | No stable model | | | | | | | | | | | | | *≥1 cognitive BS × ageb* | No stable model | | | | | | | | | | | | | | | |
| *≥1 cognitive BS- novelty × age^b^* | No stable model | | | | | | | | | | | | | *≥1 cognitive BS- novelty × age^a,b^* | No stable model | | | | | | | | | | | | | | | |
| *≥1 cognitive BS- frequency × age,b* | No significant interaction | | | | | | | | | | | | | *≥1 cognitive BS- frequency × ageb* | 0.058 | | | 0.023 | | 6.112 | | | 0.003 | | 1.059 | | 1.0-1.1 | | χ^2^_(1)_=5.291, p=0.021 | |
| **EFFECT ON PRESENCE OF LOW FUNCTIONING** | | | | | | | | | | | | | | | | | | | | | | | | | | | | | | |
| Age group 8-19 yearsa | | | | | | | | | | | | | | Age group 20-40 years | | | | | | | | | | | | | | | | |
| *≥1 cognitive BS × ageb* | No stable model | | | | | | | | | | | | | *≥1 cognitive BS × agec* | | No stable model | | | | | | | | | | | | | | |
| *≥1 cognitive BS- novelty × age^b^* | No stable model | | | | | | | | | | | | | *≥1 cognitive BS- novelty × age^d^* | | 0.054 | | 0.023 | 6. 707 | | | 0.017 | | | 1.056 | | 1.0-1.1 | | | χ^2^_(1)_=4.301, p=0.018 |
| *≥1 cognitive BS- frequency × ageb* | 0.107 | | 0.047 | | 5.201 | | 0.012 | | 1.113 | 1.0-1.2 | | χ^2^_(1)_=3.876, p<0.001 | | *≥1 cognitive BS- frequency × ageb* | | 0.100 | | 0.026 | 14.431 | | | 0.001 | | | 1.106 | | 1.1-1.2 | | | χ^2^_(1)_=10.456, p<0.001 |

Binary logistic regression analyses with method ‘backward’ and ‘forward’ using age (in years), the respective criterion requirement and their interaction term as independent

variables. Only stable models are reported (i.e., both methods revealed significant interaction effects). Otherwise, ‘no stable model’ or ‘no interaction effect’ is stated. Age entered as a continuous variable.

a Interaction became significant when all 12 cognitive BS were considered: 12 cognitive BS-novelty on axis-I disorders: Exp(β)=1.029; 95%CI: 1.0-1.1; p=0.033), 12 cognitive BS on functioning: Exp(β)=1.047; 95%CI: 1.0-1.1; p=0.017)..

b Result remained stable when the age threshold was set at 25 years, i.e. age groups were 8-24 years and 25-40 years, respectively.

c Additionally, age became significant and entered the model: perceptive BS: Exp(β)=1.272; 95%CI: 1.0-1.6; p=0.017), perceptive BS-novelty: Exp(β)=1.272; 95%CI: 1.0-1.6; p=0.017)., perceptive BS-frequency: Exp(β)=1.270; 95%CI: 1.0-1.6; p=0.018)

| **Perceptive basic symptoms (BS)** | | | | | | | | | | | | | | | | | | | | | | | | | | | | | | | | | |  |
| --- | --- | --- | --- | --- | --- | --- | --- | --- | --- | --- | --- | --- | --- | --- | --- | --- | --- | --- | --- | --- | --- | --- | --- | --- | --- | --- | --- | --- | --- | --- | --- | --- | --- | --- |
|  | β | SE | Wald (df=1) | | p after bootstrap | | Exp (β) | | 95% CI | | Omnibus Test | | |  | | | β | SE | | | Wald (df=1) | | | p after bootstrap | | | Exp (β) | | | 95% CI | | Omnibus Test | | |
| **EFFECT ON PRESENCE OF AXIS-I DISORDER** | | | | | | | | | | | | | | | | | | | | | | | | | | | | | | | | | |  |
| Age group 8-17 years | | | | | | | | | | | | | Age group 18-40 years | | | | | | | | | | | | | | | | | | | | |  |
| *≥1 perceptive BS × agec* | 0.102 | 0.039 | | 6.669 | | 0.004 | | 1.107 | | 1.0-1.2 | | χ^2^_(2)_=13.872, p<0.001 | *≥1 perceptive BS × age* | | No significant interaction | | | | | | | | | | | | | | | | | | |  |
| *≥1 perceptive BS- novelty × age^c^* | 0.102 | 0.039 | | 6.669 | | 0.001 | | 1.107 | | 1.0-1.2 | | χ^2^_(2)_=13.872, p<0.001 | *≥1 perceptive BS- novelty × age* | | 0.085 | | | 0.019 | | 20.263 | | | 0.001 | | | 1.089 | | | 1.0-1.1 | | χ^2^_(1)_=19.915, p<0.001 | | |  |
| *≥1 perceptive BS- frequency × agec* | 0.151 | 0.060 | | 6.423 | | 0.002 | | 1.163 | | 1.0-1.3 | | χ^2^_(2)_=14.732, p=0.001 | *≥1 perceptive BS- frequency × age* | | No significant interaction | | | | | | | | | | | | | | | | | | |  |
| **EFFECT ON PRESENCE OF LOW FUNCTIONING** | | | | | | | | | | | | | | | | | | | | | | | | | | | | | | | | | |  |
| Age group 8-17 years | | | | | | | | | | | | | Age group 18-40 years | | | | | | | | | | | | | | | | | | | | |  |
| *≥1 perceptive BS × age* | No stable model | | | | | | | | | | | | *≥1 perceptive BS × age* | | | No stable model | | | | | | | | | | | | | | | | | |  |
| *≥1 perceptive BS- novelty × age* | No stable model | | | | | | | | | | | | *≥1 perceptive BS- novelty × age* | | | No significant interaction | | | | | | | | | | | | | | | | | |  |
| *≥1 perceptive BS- frequency × age* | No stable model | | | | | | | | | | | | *≥1 perceptive BS- frequency × age* | | | 0.128 | | | 0.056 | 5.203 | | 0.001 | | | 1.137 | | | 1.0-1.3 | | | | | χ^2^_(1)_=5.669, p=0.017 |  |

Binary logistic regression analyses with method ‘backward’ and ‘forward’ using age (in years), the respective criterion requirement and their interaction term as independent

variables. Only stable models are reported (i.e., both methods revealed significant interaction effects). Otherwise, ‘no stable model’ or ‘no interaction effect’ is stated. Age entered as a continuous variable.

a Interaction became significant when all 12 cognitive BS were considered: 12 cognitive BS-novelty on axis-I disorders: Exp(β)=1.029; 95%CI: 1.0-1.1; p=0.033), 12 cognitive BS on functioning: Exp(β)=1.047; 95%CI: 1.0-1.1; p=0.017)..

b Result remained stable when the age threshold was set at 25 years, i.e. age groups were 8-24 years and 25-40 years, respectively.

c Additionally, age became significant and entered the model: perceptive BS: Exp(β)=1.272; 95%CI: 1.0-1.6; p=0.017), perceptive BS-novelty: Exp(β)=1.272; 95%CI: 1.0-1.6; p=0.017)., perceptive BS-frequency: Exp(β)=1.270; 95%CI: 1.0-1.6; p=0.018).

**Online Resource 6** Interactions of age and presence of ≥1 basic symptom (BS) parameter significantly impacting on the proxy measures of clinical significance, i.e., impaired psychosocial functioning (score <71on the Social and Occupational Functioning Assessment Scale, SOFAS) and presence of any non-psychotic axis-I disorder according to DSM-IV according to Online resource 5

Please note: Important German terms and unlabeled variable names are translated and explained, respectively, at first occurrence in the document. For immediate recognition, they are given in red.


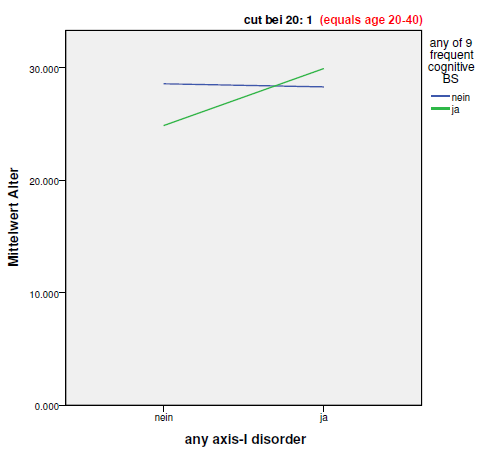

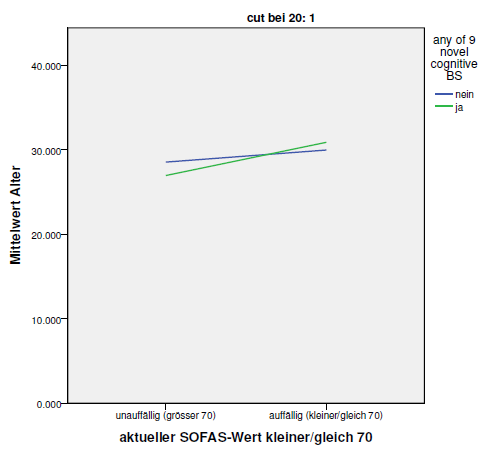

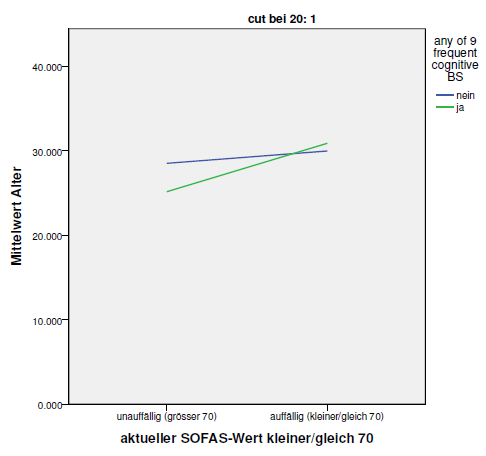


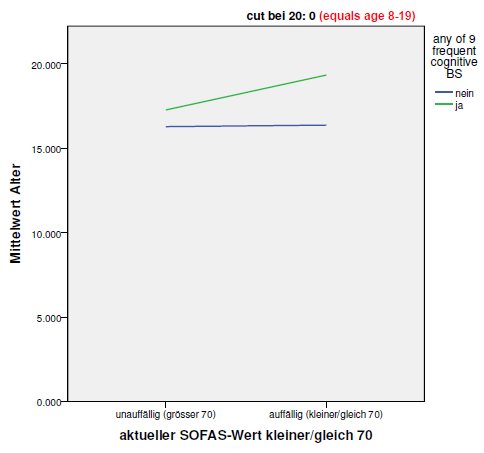


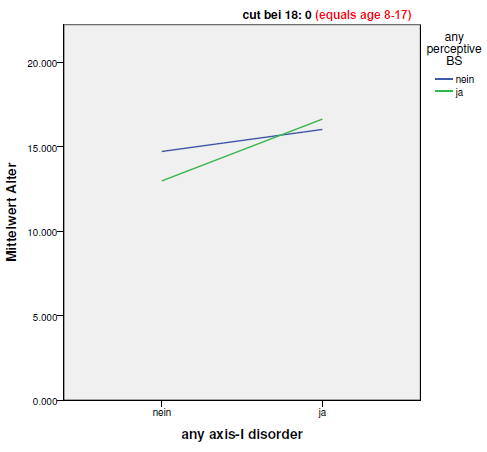

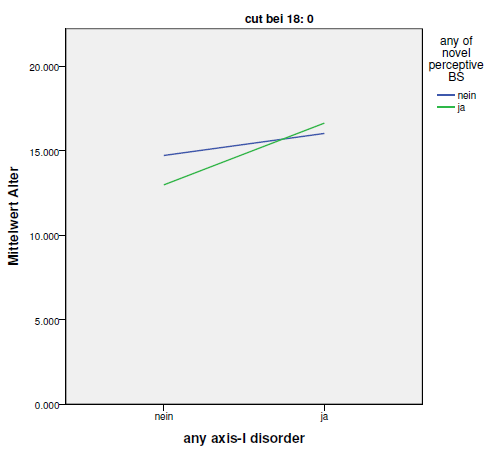

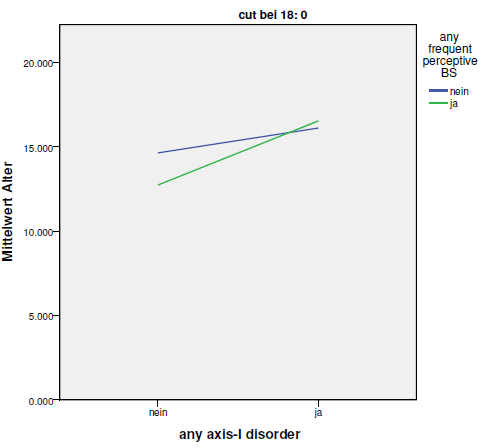


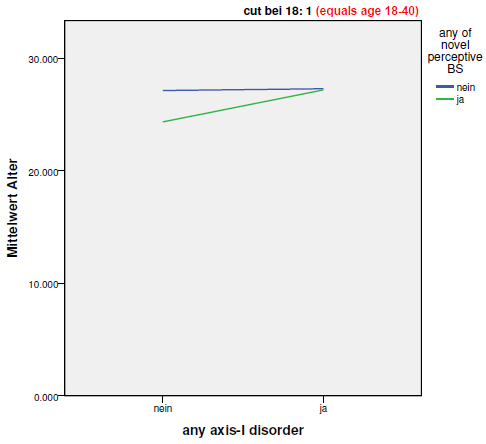

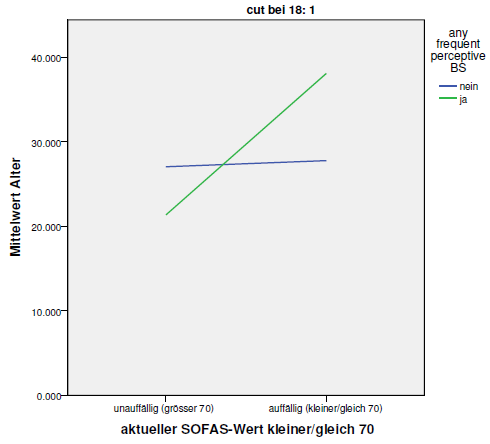

Supplement: Supplementary file 1 — Supplementary material 1 (DOCX 547 KB) [file 406_2018_949_MOESM1_ESM.docx]
